# 203. *Gardnerella vaginalis* Bacteremia in Male Patients: A Case Series and Review of the Literature

**DOI:** 10.1093/ofid/ofab466.405

**Published:** 2021-12-04

**Authors:** Christine Akamine, Shahriar Tavakoli-Tabasi, Andrew Chou, Daniel M Musher

**Affiliations:** Baylor College of Medicine, Houston, Texas

## Abstract

**Background:**

**Introduction:** *Gardnerella vaginalis* is a colonizer of the female genitourinary tract and can cause serious morbidity as a pathogen. It is an uncommon cause of infection in men and bacteremia with this organism is rare. We describe two cases of *G. vaginalis* bacteremia in male patients. A literature search was performed for cases of *G. vaginalis* bacteremia in men. A total of 13 patients were identified and discussed.

**Methods:**

**Case 1:** A 52-year-old man with diabetes and prior nephrolithiasis presented for dysuria, hematuria, and left sided flank pain. He was febrile and tachycardic with mild left costovertebral angle tenderness, leukocytosis and acute kidney injury. Urinalysis revealed pyuria. Computed tomography of the abdomen and pelvis showed pyelonephritis and a small calculus of the proximal left ureter. He was treated with ceftriaxone and then piperacillin-tazobactam. Aerobic culture of the urine yielded < 10,000 cfu/mL of mixed gram-positive flora. Blood cultures yielded *G. vaginalis* after 48 hours. He was treated with ciprofloxacin 500 mg orally twice daily for 7 total days and clinically recovered. **Case 2:** A 61-year-old man with alcohol use disorder and gout, presented with altered mental status. He had leukocytosis and acute kidney injury and was treated with vancomycin and cefepime with clinical improvement. Admission blood cultures demonstrated *G. vaginalis* in the anaerobic bottle of 1 of 2 cultures, reported 96 hours after collection. Urine culture was negative. The patient was treated with amoxicillin-clavulanate on discharge to complete a 14-day course with clinical resolution.

**Results:**

see above

Gram stain of *G. vaginalis* on blood culture

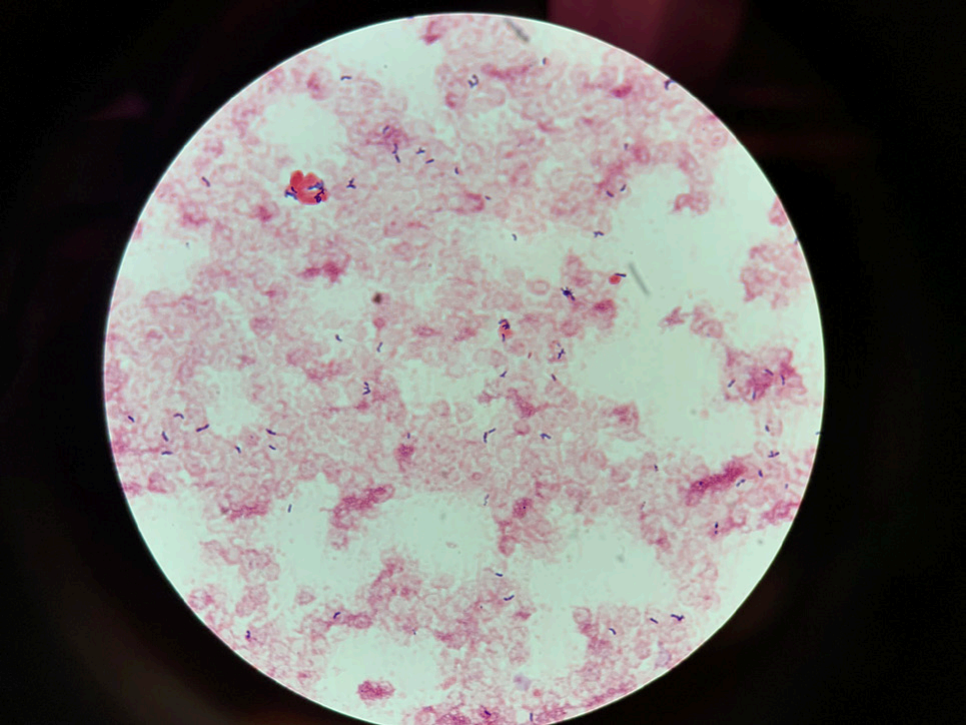

**Conclusion:**

**Discussion:** *G. vaginalis* is a facultative anaerobic gram-positive pleomorphic rod, which can be gram variable due to poor staining of the thin peptidoglycan cell wall. Isolation and identification are often delayed. Bacteremia in men is rare but nearly all have originated in the genitourinary tract. The most severe cases of *G. vaginalis* bacteremia implicate endocarditis, urethral stricture and an empyema as the sources. Collection of blood cultures and speciation are often delayed, ranging from 48 hours to 7 days. Selection and duration of treatment have ranged widely in previously reported cases, likely due to the lack of guidance regarding effective treatment.

**Disclosures:**

**Andrew Chou, MD**, **bluebird bio** (Shareholder)

